# Promising practices for the collaborative planning of integrated health campaigns from a synthesis of case studies

**DOI:** 10.1136/bmjgh-2022-010321

**Published:** 2022-12-14

**Authors:** Eva Bazant, Carol McPhillips-Tangum, Sumitra Devi Shrestha, Preetha G S, Ajay Khera, Laura Nic Lochlainn, Esmael Habtamu, Vivek I Patel, Gladys Muhire, Kristin N Saarlas

**Affiliations:** 1 Health Campaign Effectiveness Coalition, The Task Force for Global Health, Decatur, Georgia, USA; 2 CMT Consulting LLC, Decatur, Georgia, USA; 3 Health Education Agriculture and Logistics Group, Kathmandu, Nepal; 4 International Institute of Health Management Research, Delhi, India; 5 EngenderHealth, Delhi, India; 6 Department of Immunization, Vaccines & Biologicals (IVB), World Health Organization, Geneve, Switzerland; 7 International Centre for Eye Health (ICEH), London School of Hygiene & Tropical Medicine, London, UK; 8 Catholic Relief Services, Baltimore, Maryland, USA

**Keywords:** Child health, Control strategies, Health systems, Immunisation, Nutrition

## Abstract

A combination of public health campaigns and routine primary healthcare services are used in many countries to maximise the number of people reached with interventions to prevent, control, eliminate or eradicate diseases. Health campaigns have historically been organised within vertical (disease-specific) programmes, which are often funded, planned and implemented independently from one another and from routinely offered primary healthcare services. Global health agencies have voiced support for enhancing campaign effectiveness, including campaign efficiency and equity, through collaboration among vertical programmes. However, limited guidance is available to country-level campaign planners and implementers about how to effectively integrate campaigns. Planning is critical to the implementation of effective health campaigns, including those related to neglected tropical diseases, malaria, vitamin A supplementation and vaccine-preventable diseases, including polio, measles and meningitis. However, promising approaches to planning integrated health campaigns have not been sufficiently documented. This manuscript highlights promising practices for the collaborative planning of integrated health campaigns that emerged from the experiences of eight project teams working in three WHO regions. Adoption of the promising practices described in this paper could lead to enhanced collaboration among campaign stakeholders, increased agreement about the need for and anticipated benefits of campaign integration, and enhanced understanding of effective planning of integrated health campaigns.

**Summary box**

- Global health organisations have called for increasing cross programme or intersectoral collaboration to promote health campaign effectiveness, efficiency and equity; however, little has been documented about the promising approaches for planning integrated health campaigns, especially those related to neglected tropical diseases, malaria, vitamin A supplementation and vaccine-preventable diseases.
- Ten promising practices for the collaborative planning of integrated health campaigns were identified from a synthesis of eight case studies in six countries. In the campaign phase of preplanning, promising practices emerged related to coordinating bodies, securing broad participation at all levels, decision-making and pairing a campaign intervention with another familiar campaign. In the planning phase, promising practices emerged related to monitoring readiness, adopting digital tools, ensuring community acceptability and identifying missed populations. In the planning phase, promising practices were identified related to harmonising tools and setting up campaign workers for success.
- Country-based campaign planners and implementers, government health programmes, campaign funders, global institutions and non-governmental organisations can put into action these promising practices and other approaches to work towards a strategic balance of health campaigns and ongoing services for delivery of lifesaving interventions, shifting away from exclusively vertical (disease-specific) campaign approaches towards those that promote synergies and optimise efficiency, effectiveness and equity across health programmes and other sectors through enhanced coordination and collaboration.

## Introduction

Many countries rely on a combination of ongoing health services and public health campaigns to extend the reach of interventions designed to prevent, control, eliminate or eradicate diseases. Public health campaigns are time-limited, targeted and intermittent activities deployed to address specific epidemiological challenges, fill delivery gaps or provide surge coverage for health interventions. Campaigns are used to control and treat neglected tropical diseases (NTDs) and malaria, provide vitamin A supplementation (VAS) and/or prevent and address outbreaks of vaccine-preventable diseases. More than 450 health campaigns have been planned worldwide each year since 2020.^1^ The costs associated with health campaigns are considerable, with an analysis suggests that funders direct nearly US$7 billion annually towards these five priority programme areas.^2^ Campaign costs are estimated to be at least US$2.1 billion.^2^

Health campaigns have historically been organised within vertical (disease-specific) programmes, often funded, planned and implemented independently from one another and from primary healthcare services. In settings where multiple campaigns occur, planning and implementation may be carried out with little communication or collaboration among campaigns and with inadequate coordination with country health systems. Strategic and operational inefficiencies and inequities may result, which strain health systems, burden healthcare workers, weaken health services and limit campaigns’ potential health impact.^3^

There is an increasing recognition that collaborative or integrated approaches to campaign planning and implementation could increase their effectiveness, efficiency and equity.^3^ Global health agencies have voiced support for collaboration among vertical programmes. The WHO, the Global Polio Eradication Initiative, UNICEF and Gavi, the Vaccine Alliance, have each recently released guidance promoting campaign approaches that shift away from exclusively vertical programmatic approaches towards those that promote synergies and optimise efficiency through enhanced coordination and collaboration.^4–9^

There are varying types of integration that programmes can undertake.^10^ Full integration involves coordinating most or all typical campaign components (eg, microplanning, household registration, logistics, implementation and distribution, evaluation) to codeliver or simultaneously offer two or more health interventions at the point of delivery. Partial integration involves the collaboration or sharing of campaign components between vertical health programmes to improve efficiency and effectiveness of multiple campaigns, but without codelivery of interventions at the same service delivery points.^10–12^ Planning a fully or partially integrated campaign is a complex and collaborative process that requires input from multiple stakeholders covering different health programmes and across the global, national, regional and local levels of governments and implementing partners. Frameworks exist to describe the dynamics of collaborative planning and governance in health.^13–18^ However, despite the fact that planning is the foundation of effective health campaigns, little is documented on the planning of integrated health campaigns, especially those related to NTDs, malaria, VAS and vaccine-preventable diseases, including polio, measles and meningitis. This manuscript describes a novel effort to identify and document promising practices for collaborative planning of integrated health campaigns emerging from eight case studies.

## Engagement of country partners

The Health Campaign Effectiveness (HCE) Coalition, launched in 2020 by the Task Force for Global Health, fosters learning and systems change related to health campaigns and brings together country leaders, donors, multilateral organisations, non-governmental organisations and specialists working in programmes operating health campaigns in NTDs, malaria, VAS and vaccine-preventable diseases, including polio. The HCE Coalition’s research and learning agenda, developed in consultation with a committee of scientific and technical advisors, articulated the need to study opportunities, identify promising practices and document outcomes of integrated campaigns.^19^ In November 2020, HCE sought proposals from countries to identify, support and document case studies of collaborative planning approaches for integrated health campaigns. The funding criteria included that organisations needed to work closely with government agencies on integrated health campaign planning, complete the projects in 6 months and within a limited budget, and focus on integrated campaigns related to NTDs, malaria, VAS or vaccine-preventable diseases. Project funds were approved for convening stakeholders and collecting data to answer implementation research questions.

In 2021, projects meeting the criteria were selected in six countries in the Americas, Africa and Southeast Asia. Each project team tailored the case study to their own country context and needs.^20^ Table 1 provides information about location, focus, objectives and methods used in each of the eight case studies. The primary study objectives spanned the need to identify enablers and barriers to collaborative microplanning; identify training and supervision needs of health workers, explore the acceptability of collaborative approaches to supply chain planning, logistics and management; and assess the feasibility of shared data systems, digital tools and communication strategies.

**Table 1:** Descriptive characteristics of case study projects on collaborative planning for campaign integration

Country: project lead	Health domains	Primary study objectives*	Study methods
Colombia: Universidad de los Andes^26^	NTDs (soil-transmitted helminthiasis, trachoma, ectoparasites)	Identify integrated health promotion and disease prevention strategies to reduce the prevalence of trachoma and soil transmitted helminthiasis in the indigenous communities of the Cubiyú River, Vaupes Glean the perspectives of the community and stakeholders on NTD prevention and health promotion	Observation (203 people) and recording in a community-based, georeferenced information system, SIBACOM PLUS Eight planning workshops of 4–55 participants each with communities and workshop of stakeholders; 4 key informant interviews (KII) with authorities and disease teams
Ghana: UNICEF/Ghana Health Service^25^	Monovalent oral polio vaccine type 2 and vitamin A supplementation	Evaluate the effectiveness of campaign messages and strategies for social mobilisation Determine the coverage of Monovalent oral polio vaccine type 2 and vitamin A supplementation	Desk review of reports Review of household monitoring data (coverage verification) with randomised independent verification by monitors 18 KII from government, partners, civil society and representatives from regions and districts
Guinea: Fondation Santé et Développement Durable (CEFORPAG)^27^	Immunisations (measles and meningitis A)	Identify challenges and opportunities to collaborative micro-planning for the meningitis A-measles campaign Assess the acceptability and feasibility of and facilitators and barriers related to the integrated campaign in the context of the pandemic and multiple epidemics	Desk review of reports and KII KIIs with lead of programmes at the Ministry of Health (MOH), programme managers, technical and financial partners, health district leaders, health centre leaders, community leaders and 20 households/area
India: International Institute for Health Management and Research, Delhi^31^	NTDs (LF and soil-transmitted helminthiasis) and screening for chronic conditions	Understand experiences of stakeholders on the planning and delivery of integrated campaigns in Gorakhpur and Deoria districts Identify facilitators and barriers to integrating NTD and other health campaigns from the perspective of stakeholders involved in planning, operationalising and monitoring	21 KII at district and block levels 12 focus group discussions at block and grassroots level Survey of 247 community members in Gorakhpur and 109 from Deoria districts
India; PATH^28^	NTDs (LF, soil-transmitted helminthiasis)	Identify facilitators and barriers to operational efficiency and synergy for integrated delivery of campaigns for LF and soil transmitted helminths Utilise learnings from the Pulse Polio programme to improve an integrated campaign for NTDs	46 KII with national and state programme officers, district, block medical officers, and at village level/field-level workers
Nepal: Health, Education, Agriculture and Logistics^32^	NTDs (LF, soil-transmitted helminthiasis) and vitamin A supplementation	Explore potential benefits or challenges of integrating campaigns Initiate collaboration across programme and logistics divisions involved in LF and vitamin A campaigns and develop a plan Conduct the integrated campaign in a municipality of Lamjung district	22 KII from municipality, Provincial Health Directorate, Divisions, MOH and Population, beneficiaries 8 focus group discussion with community health volunteers
Nigeria; Clinton Health Access Initiative^30^	Immunisations (measles and meningitis A)	Document the integrated campaign preplanning and planning processes in Kwara, Kogi and Niger states Identify facilitators and barriers to integrated campaign planning Develop blueprints, models and transferable guidance from the integration process	Desk review of past campaign planning documents from Kogi, Kwara and Niger states and national level 10 KII and 7 focus group discussions with National and State Primary Health Care Development Agencies
Nigeria Ibolda Health International^33^	Malaria (seasonal malaria chemoprevention and insecticide-treated bed nets	Document the decision-making process used to establish the feasibility of conducting integrated Insecticide-treated net/seasonal malaria chemoprevention campaigns in a study of potential integration in Gombe and Jigawa states Identify relevant structures required and possible challenges that could affect the implementation of partial and full integrated campaign planning processes	91 KII (7 national, 14 state level, 66 at local government area level, 4 implementing partners)

*Study descriptions are at campaigneffectiveness.org.

KII, key informant interview; LF, lymphatic filariasis; NTDs, neglected tropical diseases.

Two projects examined recent campaigns, while the rest explored potential or planned integrated campaigns or assessed a pilot integrated campaign. Three projects addressed integration across programme areas (NTDs and vitamin A, polio vaccine and vitamin A, NTDs and screening for chronic conditions), and the rest related to integration within programme areas. The project’s data collection methods included the review of administrative and campaign data and conducting key informant interviews at national, subnational and district levels and focus group discussions with community health workers or members (table 1).

All projects required informed consent from participants prior to data collection and received approval from a local research ethics committee and followed country protocols on COVID-19. Full descriptions are available on campaigneffectiveness.org.

## Approach to identifying promising practices

Case study projects followed a case study template describing the background and approach, results, challenges to the collaborative planning and mitigation strategies, promising practices, lessons learnt and implications for use and application of findings to future campaigns. Case studies reports and study tools were reviewed by subject-matter experts engaged with the HCE Coalition.

A review of the literature on the definitions of and criteria for evidence based, best or promising practices informed the definition of a promising practice for this synthesis as an action that campaign planners and implementers should consider incorporating into their campaign planning processes.^21–23^ The process of analysis for the synthesis followed several steps. First, two coders reviewed each case study to extract key information and compiled an initial list of 65 project-specific promising practices in a spreadsheet. Second, using Miro, a collaborative whiteboarding tool,^24^ the initial coded practices were grouped into larger categories and informed the development of ten overarching promising practices. Third, project-specific promising practices and the overarching promising practices were presented and discussed with advisors to the HCE Coalition.

## Promising practices for the collaborative planning of integrated health campaigns

As the study team reviewed promising practices for collaborative campaign planning in the case studies, several phases of campaign planning emerged: the preplanning, planning and preparation phases. Ten overarching promising practices emerged from the synthesis of case studies in these phases, as follows:

In the preplanning phase, four practices emerged

### Facilitate participatory decision-making by forming a coordinating body to oversee campaign integration and collaborate with regional/local coordinating bodies

While coordinating bodies are commonly used in vertical campaigns, this structure, with appropriate high-level governance, takes on vital importance role in integrated campaigns, which are more complex than vertical campaigns. For example, in Ghana, UNICEF and the Ghana Health Service, described the use of a national Emergency Operations Centre, chaired by the Director of Public Health, as an intersectoral planning and implementation committee responsible for campaign planning and coordination, implementation and resource mobilisation for the integrated campaign involving polio and VAS.^25^ Subnational committees provided day-to-day monitoring and support, often in the form of reviewing daily performance and taking action to improve operations specific to integrated campaigns.

### Secure broad participation, commitment and buy-in early in the campaign planning process by engaging stakeholders at all levels, including at the national, regional/district and local/community levels

In Vaupes, a department of Colombia in the Amazonas Region, the University of the Andes worked alongside the Ministry of Health (MOH) to integrate a NTD campaign against soil-transmitted helminthiasis, trachoma and ectoparasites. The project team underscored the importance of making campaign decisions in a participatory manner and obtaining endorsement for the integrated campaign early on from multiple stakeholders—including those at the national level, in the municipal and departmental health entities, and among authorities/leaders in the indigenous communities.^26^

### Enable timely and context-specific campaigns by allowing for decentralised campaign decision-making to meet unusual conditions (eg, multiple epidemics) in certain areas, as appropriate

During the study period, Guinea was facing various outbreaks including Ebola, Marburg, measles, yellow fever and the COVID-19 pandemic. The MOH had competing priorities to which they needed to respond. Although much campaign decision-making must be done centrally, the study team Fondation Santé et Développement Durable suggested that decentralising the integrated meningitis and measles campaign decision to allow for localised decision-making in the area known as the meningitis belt would allow for activities to start and enhance efficiency.^27^

### Embrace the learnings of previously successful platforms and approaches and build acceptance of the integrated campaign by pairing the campaign with another familiar and popular campaign

In Uttar Pradesh, India, the PATH project team working with state and local government, determined that an integrated campaign for lymphatic filariasis (LF) and soil transmitted helminthiasis could achieve a greater impact by embracing the learnings from Pulse Polio Immunisation, one of India’s largest and most successful health campaigns.^28 29^ These learnings included creating opportunities for campaign activities to be reviewed at multiple levels (eg, national, subnational, community), conducting daily briefings with supervisors, field workers and officials, using interactive methods for capacity building, strengthening monitoring and evaluation, and involving relevant stakeholders for advocacy.^28^

In the planning phase, four practices emerged

### Ensure that planning for integrated campaigns assesses the readiness for campaign integration at different geographical levels

Projects described the use of readiness or preparedness assessments to assess whether a community or locale was moving towards readiness to implement an integrated campaign. For example, the Clinton Health Access Initiative (CHAI) and the National Primary Health Care Development Agency in Nigeria described efforts to redesign the readiness tools and templates used in the planning and implementation of the integrated measles and meningitis A to ensure that they work as well for integrated campaigns as they do in single intervention campaigns.^30^ In addition, the project team in Ghana assessed preparedness for campaign integration at the national, regional and district levels across campaign activities, such as planning, coordination and financing; training on supplemental immunisation activities; monitoring and supervision; vaccine, cold chain and logistics; and advocacy, social mobilisation and communication.^25^

### Facilitate supply chain and logistics management, coordination meetings, training and real-time monitoring of campaigns by using technology and digital tools

A range of technology and digital tools were used and suggested for planning, managing and coordinating of integrated campaign planning, including video conferencing, short message/text messaging, electronic dashboards, health information management systems and digitised beneficiary lists. In Ghana, the project team described using bulk short message/texting and WhatsApp to regularly share campaign messages with communities and enable campaign workers to share daily progress and address emerging challenges in real time.^25^ Use of these digital tools helped them with many campaign activities, including supply chain and logistics and data collection needed for supervision and monitoring.

### Increase community acceptability of campaign interventions by enabling the community to observe trusted leaders’ actions (eg, demonstrating taking medications) and learn from culturally sensitive information, education and communication material addressing concerns about integrated interventions

In Uttar Pradesh, India, leaders and government officials consumed antifilarial drugs in public view during the mass drug administration to dispel concerns regarding the drug.^31^ In Nepal, the project team Health Education Agriculture and Logistics (HEAL), assessing a pilot programme of partial integration of VAS and LF campaigns, developed informational materials (eg, posters) with inputs of health workers and volunteers, students and the community members to address the misconception that people with hypertension and diabetes should not take LF medicine.^32^

### Identify populations missed by traditional campaigns through nuanced strategies

In Nepal, the programme offering medicine for LF collaborated with the national VAS programme to find people who had been missed by the LF campaign.^32^ The local authorities, with support of project team HEAL, developed the complementary monitoring and supervision approach during home visits, which consists of: providing information about the two campaigns being integrated to community members; enlisting community health volunteers to ask community members whether they had taken LF medication; identifying community members who had been missed by the LF campaign; educating community members about LF; referring community members to a nearby health facility to take LF medication and reporting information to the health facility. High coverage of LF medicine and VAS (85%+) was reported in the municipality in which the pilot project was conducted.

In the preparation phase, two practices emerged:

### Meet the information and knowledge needs of the integrated campaign by harmonising tools, templates and guidance from standalone campaigns early in the campaign timeline

In Uttar Pradesh, India, PATH developed a single monitoring tool for the integrated campaign that harmonises the monitoring tools of single intervention campaigns and trained personnel in its use.^28^ In Nigeria, the project team supporting the National Malaria Elimination Programme, Ibolda Health International, noted that an electronic dashboard was a critical tool. The dashboard was useful for monitoring campaign progress and providing real-time information to support training of healthcare workers, deployment of human resources, and coordination of logistics, community mobilisation and distribution of campaign commodities.^33^

### Set up campaign workers for success by providing appropriate training, supportive supervision, incentives and recognition, and promoting the transparency and accountability needed for timely remuneration

In Uttar Pradesh, India, the International Institute for Health Management and Research (Delhi) project team described providing campaign workers with a single training manual in the local language that details the integrated campaign activities to promote a systematic and unified process of campaign delivery.^31^ The authorities in Uttar Pradesh also recognise and give awards to community health workers who deliver the integrated/vertical campaigns, in an annual event, called Accredited Social Health Activist (ASHA) Day.

These 10 promising practices are shown with illustrative tools from the projects and other partners (table 2).

**Table 2:** Overarching promising practices and illustrative tools

#	Promising practice (abbrev.)	Illustrative tools to support implementation
1	3. Form a coordinating body to oversee the integrated campaign	Process Flow: Collaborative Planning of Integrated Campaigns.^34^
2	Engage stakeholders early and at all levels	Interview Guide: Community Perceptions of Integrated Campaigns;^35^ Partner Integration Experiences Interview Guide.^36^
3	Assess readiness for campaign integration at all levels	Preparedness Assessment Checklist;^37^ Campaign Implementation Readiness Dashboard.^38^
4	Pair the integrated campaign with another familiar and popular campaign	Macroplanning Worksheet for Integrated Campaigns;^39^ Potential Campaign Integration Checklist.^40^
5	Allow decentralised campaign decision-making in unusual situations	Role of the polio network in COVID-19 vaccine delivery and essential immunisation.^41^
6	Harmonise tools, templates and guidance from vertical campaigns	Monitoring Tool for Integrated Campaigns;^42^ Vaccination Team Microplanning Tool.^43^
7	Embrace digital tools for logistics and supply chain management	Using Digital Technologies for Real-Time Monitoring of Supplementary Immunisation) Activities;^44^ Improving Insecticide-treated Net Campaign Efficiency Through Use of Digital Tools.^45^
8	Provide appropriate training, recognition and accountability to campaign workers	Guidelines for Vaccination Team Supervisors of Integrated Campaigns;^46^ Vaccination Team Guidelines Template.^47^
9	Enlist the support of trusted community leaders to increase acceptance of integrated campaigns	UNICEF | WHO: Conducting community engagement for COVID-19 vaccines.^48^
10	Identify populations missed by traditional campaigns through nuanced strategies	Caregiver Awareness Survey Tool:^49^ Using Geospatial Models to Map Zero-Dose Children.^50^

In addition, barriers may exist at multiple levels, including within countries (eg, lack of coordinated planning between ministries) and among campaign funders (eg, discordant funding priorities and timelines, differential pay scales for campaign workers). The barriers experienced in the projects and the mitigation are described in online supplemental table 1).

10.1136/bmjgh-2022-010321.supp1 Supplementary data

## Conclusion

This novel effort identified promising practices for the collaborative planning of integrated health campaigns in countries. These practices emerged from a synthesis of eight case studies across different health domains and six countries. Collaborative planning of health campaigns typically involves coordination, macroplanning, stakeholder and community engagement, microplanning, supply chain and logistics management, social mobilisation and communication, appropriate engagement, training, supervision and recognition of health workers, and monitoring. Complexity increases when each of these activities is conducted in an integrated campaign.

Research on health campaigns has typically focused on campaign outcomes (eg, coverage, access), but the appropriate planning of health campaigns—especially integrated campaigns—is foundational to their success. For this reason, it is important to identify promising practices that can help countries demystify and simplify the process of starting and collaboratively planning integrated health campaigns. Adoption of some or all of the promising practices described in this paper should lead to enhanced collaboration among key campaign stakeholders, increased agreement about the need for and anticipated benefits and potential challenges of campaign integration, and enhanced understanding of effective approaches for planning integrated health campaigns.

Several actions could help countries adopt the promising practices described in this paper at the level of campaign planners, implementers and partners. Campaign planners and implementers must be made aware that an initial set of promising practices for the collaborative planning of health campaigns has been identified. The HCE Coalition has initiated is disseminating the practices, so that countries may use them and document and share their experiences with one another. In this way, we can collectively build the evidence base to guide integrated campaign planning in the coming years. Although the promising practices described in this paper should not be expected to work equally well in every setting, the fact that these practices are grounded in the experiences of projects in different countries should help assure campaign planners and implementers that these practices warrant additional testing and potential adaptation to suit their specific needs. Campaign planners and implementers will need to advocate for the adoption and adaptation of these practices. To enhance the knowledge, skills, and self-efficacy of campaign planners and implementers of advocacy, resources should be developed and made available.

Campaign partners, which include government health programmes, campaign funders, global institutions and non-governmental organisations, should coalesce around the need to adopt and adapt strategies to plan for enhanced campaign effectiveness, efficiency and equity in integrated campaigns. Campaign partner endorsement of the promising practices described in this paper would be an important first step towards this goal. Campaign partners must commit to the development and implementation of strategies to mitigate existing programmatic and financial disincentives to campaign integration. Campaign partners should support documentation of what works and what doesn’t work and invest in implementation research to develop further evidence and to move from promising to evidenced-based best practices. Campaign funders should come together with an eye towards enhanced health campaign effectiveness, efficiency, equity and country ownership. Future studies can expand and build on this initial set of practices, to move beyond planning to incorporate findings across the campaign cycle.

Authorities in countries are highly motivated to optimise the use of limited human resources and technologies and maximise the impact of their health campaigns. During the COVID-19 pandemic, government health programmes were overstretched needing to add COVID-19 vaccination campaigns to ongoing activities. The practical, experience-based collaborative planning practices described in this paper will enable country partners and campaign-interested stakeholders to shift towards approaches that promote synergies through enhanced coordination and collaboration to increase campaign effectiveness.

## Data Availability

Data are available in a public, open access repository. The eight case studies are available at: https://campaigneffectiveness.org/case-studies-on-integrated-health-campaigns/
