# Supplementary material for: Promising practices for the collaborative planning of integrated health campaigns from a synthesis of case studies
Source: BMJ Glob Health. 2022 Dec 14;7(12):e010321. doi: 10.1136/bmjgh-2022-010321 (PMC9756207; doi:10.1136/bmjgh-2022-010321)
Supplement: Supplementary data [file bmjgh-2022-010321supp001.pdf]

Supplemental Table 1. Barriers and Mitigation Related to Integrated Health Campaign Planning in Projects Supported by the Health Campaign Effectiveness Coalition

Project	Challenges/Barriers Faced	Mitigation
Colombia/ Universidad de los Andes [21]	• Socio-cultural beliefs about infectious diseases and treatment<br>• Language barriers (indigenous languages)<br>• Remote locations/travel cost (many hours via boat)<br>• Demand for services in non-study communities<br>• COVID-19: delays for travel & administration	• Hired local indigenous health promoter<br>• Using microplanning for engaging with the community (knowledge; implementation)<br>• Involving local stakeholders (communication)<br>• Involve non-study communities from early planning phase
Ghana/ UNICEF/ Ghana Health Service [22]	• Late-stage decision to integrate campaigns (there was pre-campaign messaging for only 1 intervention)<br>• Refusal in community since some people thought COVID vaccine was included<br>• Sporadic shortages of vitamin A, supplies<br>• Heavy rainfall disrupted the campaign<br>• Security issues (escorts, additional team)<br>• Delay in disbursement of funds<br>• Low coverage of vitamin A (age confirmation)<br>• Children missed (absent/refused/ not visited)	• Intensified social mobilization and communication with key community leaders to address fears that the integrated campaign included COVID-19 vaccine<br>• Daily data monitoring via the Emergency Operations Center which enabled real-time identification and timely solutions<br>• A regional team provided security escort and an additional regional team<br>• Supervisors redistributed vitamin A and supplies, as needed
Guinea/ FOSAD-CEFORPAG) [23]	• Missed chances to integrate other campaigns<br>• Delays from funding, administrative procedures from new Ministry of Health Program<br>• Vaccinators dissatisfied (workload, poor motivation, administrative procedures)<br>• Community concerns (on vaccine safety, side effects)	• Prioritize integrated campaigns in most affected areas during other outbreak<br>• Decentralization of decision-making<br>• Opportunities to express concerns/ideas during pre-planning (vaccinators, communities)<br>• Timely remuneration, sufficient training, and manageable workloads of vaccinators<br>• Community sensitization to mitigate fears or adverse events.
India/ IIHMR) [24]	• COVID-19 & natural disasters caused delays<br>• Different understanding of integration<br>• Dependence on overburdened community-level cadre called ASHAs for service delivery (concern about decreased/delayed incentives)<br>• Inadequate training for ASHAs<br>• Sporadic community resistance to campaign components (antifilarials).	• Campaigns tailored to local needs, resources Integration process was allowed to vary across campaigns and districts<br>• Capacity building of the districts for needs assessment, campaign implementation<br>• Earlier planning of campaign + pilot campaign<br>• Systematic training of ASHAs; single training manual in local language<br>• ASHA: technical support, real time supervision, security, incentives.<br>• Campaign monitoring, delivery evaluation

India/ PATH [25]	COVID-19 pandemic made face-to-face interviews difficult.<br>Long approval processes.<br>Diverse ideas of the concept of integration.<br>Competing priorities (COVID vaccine)	Most interviews were virtual.<br>Face-to-face interviews with safety precautions<br>Sensitized participants on integration, prepared uniform definition through interview.<br>Regular communication/coordination with participants.
Nepal/ HEAL [26]	Competing priorities (COVID-19)<br>Officials' schedules make it difficult for planning meetings<br>Lack of policy, guidelines for integration<br>Difficulties managing integrated logistics<br>No budget for awareness-raising activities<br>Confusion among community volunteers called FHCVs in sharing information (differences in the ages for the two interventions (vitamin A and lymphatic filariasis medication, different reporting structures)<br>Drug hesitancy in the community<br>Difficulty swallowing pills (among children, disabled)	Focus on integrated project plan development<br>Collaborative planning to identify solutions<br>Continued communication with officials<br>Rescheduled LF campaign enabled use of 'complementary monitoring and supervision' approach<br>Developed and distributed local, culturally-sensitive information and education materials<br>Tablet swallowing per WHO recommendations
Nigeria/ CHAI [27]	Incomplete identification and involvement of stakeholders in planning of an integrated immunization campaign<br>Poor management of conflicting priorities<br>Delayed funding (mobilization, logistics), microplanning (population targeting, resources)<br>COVID-19 impacted planning, implementation	Regular coordination platform/forum (clear terms, aims, responsibilities)<br>Targeted social mobilization, timely advocacy (integrated decision-making)<br>Clear processes, procedures, communication (national, state, delivery-level)<br>Pre-planning for: security, transportation, infection prevention and control<br>Use virtual tools for planning meetings
Nigeria/ Ibolda Health [28]	Confusion over the concept of integration<br>Inadequate federal, state and local government area funding, over-dependence of donors<br>Concurrent health interventions used same pool of personnel<br>Insecurity, communal clashes across regions<br>Uncertainty of shipping and quality assurance and duration for insecticide treated nets<br>Drug administration schedule lacked flexibility for net distribution (inability to delay one intervention for another)<br>Different target age groups for interventions (more time, remuneration)	Sensitizing all players on benefits of integration<br>Advocacy to political leaders (at all levels), existence of a government counterpart fund and fund releases<br>Early planning, approval of campaign at all levels<br>Notifying relevant security agents on campaign<br>Alignment of personnel remuneration and campaign days<br>Development of integrated national campaign guideline<br>Ownership of the integration process by the State Primary Health Care Development Agency
